# Community perspectives on addressing and responding to HIV-testing, pre-exposure prophylaxis (PrEP) and post-exposure prophylaxis (PEP) among African, Caribbean and Black (ACB) people in Ontario, Canada

**DOI:** 10.1186/s12889-022-13093-0

**Published:** 2022-05-07

**Authors:** Josephine Etowa, Wangari Tharao, Lawrence Mbuagbaw, Shamara Baidoobonso, Ilene Hyman, Suzanne Obiorah, Muna Aden, Egbe B. Etowa, Akalewold Gebremeskel, Medys Kihembo, LaRon Nelson, Winston Husbands

**Affiliations:** 1 School of Nursing, Faculty of Health Sciences, University of Ottawa, Ottawa, Ontario Canada; 2 Women’s Health in Women’s Hands Community Health Centre, Toronto, Ontario Canada; 3 Department of Health Research Methods, Evidence and Impact, McMaster University, Hamilton, Ontario Canada; 4 Department of Community Health and Epidemiology, Dalhousie University, Halifax, Nova Scotia Canada; 5 Dalla Lana School of Public Health, University of Toronto, Toronto, Ontario Canada; 6 Suzanne Obiorah, Community and Social Services, Ottawa, Ontario Canada; 7 Canadians of African Descent Health Organization, Ottawa, Ontario Canada; 8 School of Nursing, Yale University, New Haven, CT USA

**Keywords:** HIV testing, PREP, PEP, African, Caribbean, Black (ACB) populations, Community-based participatory research (CBPR), Health providers, Qualitative research

## Abstract

**Background:**

The African, Caribbean, and Black (ACB) population of Ontario, Canada is comprised of individuals with diverse ethnic, cultural and linguistic backgrounds and experiences; some of whom have resided in Canada for many generations, and others who have migrated in recent decades. Even though the ACB population represents less than 3.5% of the Canadian population, this group accounts for 21.7% of all new HIV infections. It is well-documented that ACB populations, compared to the general population, experience multi-level barriers to accessing appropriate and responsive HIV services. In this paper, we present qualitative findings on the ACB population’s experiences with HIV-testing, pre-exposure prophylaxis (PrEP) and post-exposure prophylaxis (PEP) and obtain their perspectives on how to improve access.

**Methods:**

We conducted twelve Focus Group Discussions (FGDs), within a two-day World Café event and used socio-ecological framework and community-based participatory research (CBPR) approaches to guide this work. We meaningfully engaged ACB community members in discussions to identify barriers and facilitators to HIV testing, PEP and PrEP and how these may be addressed. The FGDs were transcribed verbatim and thematic analysis guided data interpretation. Credibility of data was established through data validation strategies such as external audit and peer-debriefing.

**Results:**

Our analyses revealed multi-level barriers that explain why ACB community members do not access HIV testing, PEP and PrEP. Fear, health beliefs, stigma and lack of information, were among the most frequently cited individual- and community-level barriers to care. Health system barriers included lack of provider awareness, issues related to cultural sensitivity and confidentiality, cost, and racism in the health care system. Participants identified multi-level strategies to address the HIV needs including community-based educational, health system and innovative inter-sectoral strategies.

**Conclusion:**

CBPR, co-led by community members, is an important strategy for identifying the multi-level individual, interpersonal, community, institutional and structural factors that increase HIV vulnerability in ACB communities, notably anti-Black systemic racism. Study findings suggest the need for targeted community-based strategies and strategies aimed at reducing health system barriers to testing and care.

## Background

The African, Caribbean, Black (ACB) population is comprised of Black Canadians of diverse ethnic, cultural and linguistic backgrounds and experiences; some of whom have resided in Canada for many generations, and others who have migrated in recent decades. In 2016, 55.7% of Canada’s ACB population were immigrants or permanent residents. More than half of Canada’s ACB population (52.4%) resided in Ontario [1].

The number of new HIV diagnoses in Canada has been relatively stable over the last two decades, with Ontario accounting for the highest number and proportion of reported cases (*n* = 881/2344 in 2016, 37.6%) [2]. Although ACB communities represent approximately 3.5% of Canada’s population, in 2016 they represented an estimated 21.9% of new diagnoses across Canada [2]. Within ACB communities, heterosexual contact accounts for approximately one-third of 33% of new HIV infections [3]. ACB communities are less likely than other racialized groups to be aware of their HIV seropositive status [4, 5], and when they are, they are typically at advanced stages of infection at the time of diagnosis [6]. It is well-documented that ACB populations, compared to the general population, experience multi-level barriers to accessing appropriate and responsive health services [7–9]. These include, discrimination, poor representation of ACB community members among health care personnel, especially at the organizational leadership and policy-making levels, lack of culturally appropriate information in relevant languages, lack of culturally competent health professionals, and lack of translation services [10–12]. These same barriers deter ACB community members from accessing appropriate and responsive HIV testing and treatment services [13, 14].

Current research demonstrates that pre-exposure prophylaxis (PrEP) is an effective intervention in reducing the risk of sexual HIV transmission among people at high risk of HIV infection [15–17]. Despite its demonstrated efficacy, there continue to be challenges to PrEP access and uptake among ACB men in Ontario. A recent Ontario study of ACB men found that ACB men who have sex with women (MSW) were much less likely to accept PrEP than ACB men who have sex with men (MSM). They also found that “self-perceived risk of acquiring HIV significantly impacted willingness of men who have sex with men (MSM) to use PrEP, but was systematically under-estimated by participants.” [18]. The majority of Canadian research on awareness and willingness to use PrEP has been conducted among gay men and other MSM, with few studies looking at other at-risk populations, such as ACB populations [19].

Post-exposure prophylaxis (PEP) is a way to help prevent the transmission of HIV in an HIV-negative person who may have been recently exposed to the virus. The World Health Organisation (WHO) recommends 1 month of triple ART as PEP following HIV exposure [20], and Canadian PEP guidelines recommend that PEP should be readily available in health care settings where there may be an urgent need [21]. However, the decision to provide PEP typically lies with the healthcare provider but must adhere to policies set by senior leadership and/or public health authorities. Even less research has explored barriers and facilitators of PEP access in different population groups [22].

### Study aim

Understanding the lived experiences and determinants of health of ACB people is recognized as key to tackling the root causes of HIV/AIDS and providing effective and specific HIV/AIDS services [23]. This paper presents the qualitative findings of a recent mixed-methods study, which explored ACB community members’ lived experiences with accessing HIV testing, PEP and PrEP in Ontario, and community perspectives on how identified barriers may be addressed.

## Methods

The study was guided by established theoretical and conceptual frameworks, a community-based participatory research (CBPR) approach, the socioecological model (SEM) and an intersectionality lens. The socioecological model [24], later adapted by Baral et al. [25], was used to identify the vast array of layered *macro-, meso- and micro-level* factors to consider when identifying barriers and facilitators to HIV care. Research shows that marginalized social identities and inequities such as sexism, racism, and homo/transphobia, overlap and simultaneously interact with individual-, community- and structural-level factors to deter access to health care and reduce opportunities [26]. Hence, intersectionality theory was used to understand and interpret how ACB people’s social identities intersect or mutually enhance to create marginalization or privilege, and how these impact on access to HIV testing and care. CBPR is a preferred approach to working collaboratively with and through groups of people or communities affected by the issues being investigated with a goal of not only studying the issue, but also to address broader issues affecting the well-being of the community [27, 28].

The study included both quantitative and qualitative components. The quantitative component was designed to estimate HIV prevalence and facilitate an improved understanding of associated behaviors, knowledge, individual-, community- and structural- factors, and health care access and utilization among ACB people in Ontario (see Mbuagbaw et al. [29] for more details). The qualitative component was designed to provide ACB community members with an opportunity to participate in the interpretation of quantitative study findings, contribute to data collection and be involved in the formulation of recommendations. The study was approved by the following affiliated institutional Research Ethics Boards (REBs): University of Ottawa Research Ethics Board; Ottawa Public Health Research Ethics Board; Toronto Public Health Research Ethics Board; Laurentian University Research Ethics Board; and University of Toronto. All methods were carried out in accordance with relevant guidelines and regulations.

Twelve focus group discussions (FGDs) were conducted over a two-day World Café event. The World Café event was guided by seven principles as outlined in the World Café Community Foundation Creative Commons Attribution (2015): a) set the context, b) create a hospitable space, c) explore questions that matter, d) encourage everyone’s contributions, e) connect diverse perspectives, f) listen together for patterns and insights, and g) share collective discoveries. Due to COVID-19 physical distancing requirements, the World Café event was revised from an in-person round-table conversation to a two-day (July 30 and 31, 2020) virtual event using the Zoom videoconferencing platform. The event was promoted among ACB social networks using social media and a flyer with the registration link and a description of the study’s purpose and the event. Each day’s session started with presentations about the study and its results by research team members. The presentations were followed by a plenary ‘questions and answers’ period and virtual focus group discussions. The FGDs were followed by a large group report-back period where key points from the discussions were summarized and shared by each focus group’s moderator and note-taker.

In total, 107 individuals from Ottawa and Toronto, Ontario, participated in the FGDs (50 on Day 1 and 57 on Day 2). Six FGDs included participants from Ottawa and six included participants from Toronto. Eight FGDs were held in English and four in French (two from in each city). FGD participants included community members and leaders, service providers, decision-makers and other knowledge users. Given the well-recognized gaps in HIV care delivery to HIV-diagnosed Francophone ACB people in Canada [30], FGDs were conducted in English and French. Each FGD was led by a facilitator who was supported by a note-taker. All facilitators and note-takers were briefly trained in focus group methodology and provided with a bilingual (French and English) focus group discussion guide that included: information for welcoming participants and facilitating introductions, key information from the consent forms, and questions to ask during the focus group discussions (including probes). The focus group participants discussed, HIV testing and counselling and experiences with addressing basic needs on the first day. On the second day, they discussed HIV vulnerability and risk, service access and HIV-related knowledge. Each FGD was audio-recorded and transcribed verbatim. Chat box texts were noted. Taking part in this research study was completely voluntary and participants could refuse to participate entirely, refuse to answer questions or withdraw from the study. Participants were also given the opportunity to verbally consent at the start of the FGD. Every focus group participant received an honorarium of CA$30 per focus group.

FGDs were transcribed verbatim and Braun and Clarke’s [31] six steps thematic analysis process guided data interpretation. A theme is a pattern found in the information that describes and organizes the possible observations, or interpretations of phenomena identified in the data. The process began with the development of a coding framework informed by questions from the interview guides and a systematic approach that involved: (1) familiarizing with the data; (2) generating initial codes; (3) developing a coding tree to guide the coding of transcripts; (4) identifying themes; (5) reviewing, defining and naming themes; 6) interpreting the narratives and stories; and (7) producing the report – a concise, coherent, logical, and non-repetitive account supported by vivid examples. Trustworthiness of the data was guided by Lincoln and Guba’s [32] criteria for ensuring scientific rigor in qualitative research including credibility. Credibility of data was established through data validation strategies such as peer debriefing and external audit [33]. Data analysis was led by the qualitative working group and all team members. Some focus group participants had opportunity to provide feedback on the data analysis and interpretation.

## Results

The key themes from the data analyses included, individual- and health system- related barriers to HIV testing, PrEP, and PEP, and how these may be addressed through community-wide educational efforts, health policies and health provider capacity-building strategies and intersectoral approaches.

### “Being HIV-positive you lose everything” - fear, health beliefs and stigma represent prominent barriers to HIV testing

At an individual level, fear was frequently cited as reason why ACB community members didn’t access HIV testing. Several focus group participants reported that since HIV continues to be perceived as a death sentence, individuals often avoid getting tested.

*They are scared to learn what their status is because he knows that HIV it kills and then maybe that the person can’t accept living with that virus. That’s why he doesn’t even want to know, even want to talk about screening and it’s why I can say that really, it’s what makes it that our Black community doesn’t even want to go [get] tested and there is a lot that incre... that it increases every year (Female, Toronto, FG4).*

Among ACB individuals who were temporary immigrants or refugee claimants, the fear of testing extended to the fear of deportation if there is a positive test.

*So some people even think that maybe if your HIV status could be a reason for you being denied coming into Canada, which is wrong (Female, Toronto, FG3)*

*And also, fear in regard to immigration status, some people have a clause that if they are found to be HIV positive they think that they can be deported back to their home country (Female, Ottawa, FG2).*

Health beliefs were a barrier to medical help-seeking in general and to HIV services more specifically. For example, there are perceptions among ACB community members that individuals do not typically use preventive health care services, such as testing. Gender differences in help-seeking behaviours were also noted. As a focus group participant explained,

*I find it’s very hard to get your Black man to go to the doctor. I have to say that, you know. They are very … I don’t know what they’re afraid of. They have to be really really sick before they decide. Say, you know, woman on a whole, we say I have a check-up, let me go see my doctor. Maybe they have their regular check-ups, so once a year. You would never really see a man do that. He has to be sick (Female, Ottawa, FG5).*

Social norms pertaining to sexual health, mental health, sex work, and gender-based violence (GBV) also contributed to stigma and acted as a barrier to HIV-testing.

*Those social norms could be the difference between whether somebody gets tested or not. Whether someone’s direct close community is encouraging them to get tested or not (Female, Toronto, FG3).*

The stigma associated with HIV was very strong. As one focus group participant explained,

*I said what my African brothers and sisters do not understand is that when you come from the Caribbean and you test positive in the Caribbean, you lose everything. You lose your house. You lose your job. You lose everything. You don’t get anything. Whereas in Africa, there is so much mobilization and there’s so much NGOs that is run by HIV positive people that the stigma that they … people come in. They don’t get the same stigma as the people from the Caribbean; right? So I think there’s a lot of stuff we have to look at and who’s getting access and why the Caribbean people are getting left behind (Female, Toronto, FG6).*

Focus group participants spoke of community members who, after sharing their HIV status with people who were part of their support networks, were subsequently rejected and stigmatized to the extent that their basic needs could no longer be met.

*I think there’s a lot of stigma when it comes to HIV. So, there’s a lot of … because most people feel like maybe if I go and test it’s maybe, like a death sentence. But also, even among their peers, and this is from a fact, when people say, there are a few people who have come out and I think that also has been like an encouragement. So and so has tested and you know, they are living their life, but also you know, there’s a lot of stigma when it comes to the subject. (Female, Toronto, FG3).*

It was further recognized that people’s experiences varied according to their social locations. Several focus group participants noted that ACB communities are extremely diverse in terms of their economic, political and social identities including, socio-economic status, education, immigration experiences, countries of origin, faith and language fluency, etc.

### “A love-hate relationship with health care” - health system barriers to HIV-testing abound

Several focus group participants expressed concerns about racism and the lack of cultural competency within health systems. For example, one participant raised the issue that many providers do not understand the lived experiences of their ACB patients.

*I have a love-hate relationship with health care. I personally don’t go to emergency and I don’t go to drop-in clinics. I have to see doctors I know. Because we have a lot of existing trauma, whether it’s trauma that’s generational trauma from you know, colonialism, we have a lot of trauma from … other Black people have a lot of trauma from other things. But trauma from even migrating to this country. And sometimes … and abuse and other things and when you go to seek health care and you start explaining this to people who don’t really understand where you’re coming from, it’s challenging (Female, Toronto, FG6).*

Several focus group participants described challenges in accessing a regular health care provider. Even when ACB community members had a regular health care provider, the lack of HIV awareness among health providers was a recurring theme. As one focus group participant explained,

*So I think there’s a lot of education that needs to be done with primary care physicians and people who are offering-- or doing the tests themselves. I think that that community, the physicians, need additional opportunities for education themselves because they’re not-- they may not be exactly aware of what’s available (Female, Ottawa, FG5).*

Some participants described being encouraged to go for testing based on their social location and identity, and others being denied testing based on the judgements of health care providers. Other focus group participants described the process of testing as degrading and insensitive. For example, participants described having to repeatedly explain and prove that they had certain symptoms and why testing was needed.

*Is it respectful to ask a third age (‘senior’) person how many sexual partners he has... why not take care of him instead of asking him this kind of question? Why ask me the reasons why I want screening tests every six months? This question is inappropriate. If I want to do screenings regularly, it’s my problem, full stop? (Moderator, Ottawa, FG4)*

Focus group participants identified confidentiality as a major concern when going for HIV testing. Several participants disclosed that they were not informed about testing prior to it being carried out and that confidentiality was essential to ensure emotional, physical and social safety.

Several focus group participants named systemic racism as a key barrier to HIV care. As one participant explained:

*Unless we start addressing systemic racism in a way that is actually effective, the stigma piece around HIV testing with ACB community will always be there (Male, Ottawa, FG2).*

Compared to the United States and other countries, focus group participants believed there were fewer HIV testing options available in Canada.

*You know, Canada is really behind when it comes to testing. In the States and other countries there’s just much more options of testing, you know, and they’re much used to going to gay prides where there’s HIV testing done on location and things like that. You know, the notion that it’s 2020 and it’s only done in certain locations or certain times, it’s really antiquated (Male, Ottawa, FG3).*

Several focus group participants recommended that testing be mandatory and/or part of a systematic health assessment, rather than optional or voluntary. As one focus group participant explained,

*With education if people know that this is something that is standardized when they come to Canada, be it immigrants or second generation or third generation knowing that this is part of the standards of keeping them healthy will make the test much more accessible and less difficult to access considering the cultural considerations and the fear and stigma that can often be associated with that (Moderator, Ottawa, French, FG1).*

However, other focus group participants expressed concerns about the time commitment involved with increasing the frequency of HIV testing and felt that testing should be associated with risk level, and not be mandatory.

*I think you should think about your own risk factors but also I think it should just be whenever you feel like you want to be tested, I don’t think there is anything like over testing. So long as you feel like maybe you’re unsafe or you feel you’re exposed or you just feel like ‘oh, no, now at this time I want to know my status’. Maybe you went and tested three months ago, I mean, a week and they tell you come back after six months and then you go back (Female, Toronto, FG3).*

Overall, many focus group participants were in favour of self-testing kits to reduce the stigma of HIV testing. As one participant explained,

*I think self-testing kits would be very helpful as far as HIV testing is concerned. There’s a general sense of fear still because of stigma that comes with HIV, even in a situation where anonymity is guaranteed. And so having a self-test, it can’t get any better than that (Male, Ottawa, FG3).*

### “There is PrEP but who can access PrEP?” – access to information about PrEP is limited

At the individual level, accessibility issues such as lack of information and location were identified. Many focus group participants were not aware or did not have direct experience with the use of PrEP/PEP treatments. One of the main reasons why these treatments are not better known in ACB communities is because they were originally marketed as a *drug for white gay men*.

*So even when we go to PrEP, which is the pre-exposure, it’s supposed to be as gay men say, gay White people say, it’s supposed to be like birth control. You take it. You don’t get pregnant (Female, Toronto, FG6).*

*There is PrEP but who can access PrEP? Who is PrEP for? How many Black people access PrEP? How many Black people know about PrEP? How many Black people can access PrEP even if they know about it? What are the questions Black people will be asked? One of my doctor friends runs a PrEP clinic. And every time he tells me I don’t see any Black men. I don’t see gay Black men (Female, Toronto, FG6).*

Focus group participants also noted that very little research has been conducted on the use of these treatments in ACB communities, and even less research has been conducted with women, which may deter Black women from feeling like this is something that they can inquire about for themselves. As a female focus group participant went on to say,

*When we look at the HIV landscape and everything, are Black women disproportionately affected by HIV more than other women? And there has never been proper research to even look at side effects of PrEP for women, PrEP and pregnancy, PrEP and all these things. So PrEP is not even for Black women (Female, Toronto, FG6).*

### “Just give it to me because you just believe that I need it” - health system barriers to PrEP/PEP are common

Focus group participants noted that while these treatments are very effective, they are not covered by public health insurance, and private health insurance plans are very costly, so these treatments are not affordable to many people.

Focus group participants who accessed PrEP treatment described experiences of feeling stigmatized when requesting this treatment. As in the case of HIV testing, ACB community members resented the need to continually prove their need for treatment.

*Don’t ask me for ID. Don’t ask me for health card. Just give it to me because you just believe that I need it. I wouldn’t come here if I didn’t need it. I think that would only … that would help in terms of how we as Black people access PrEP (Female, Toronto, FG6).*

*Even to access that PrEP you have to go and disclose … and stigma is a big issue. Because if I have to go and say I want PrEP, it means I am disclosing my partner’s status. People don’t want to do that. Because of stigma, a lot of people even when there is a discordant relationship, it is top secret. They won’t tell anybody else because if anybody else knows, it’s going to be a whole conversation and the stigma and everything. (Female, Toronto, FG6).*

Focus group participants identified numerous multi-level strategies to address HIV needs and reduce barriers to testing and care, including community-based, educational, health provider, organizational and innovative, inter-sectoral strategies.

### “Okay, this is an issue we have in the community, everyone let’s get involved” - community-wide educational efforts are needed

The need for community-wide educational efforts was identified as a way to increase to address stigma, increase individual and community HIV critical literacy about risk, prevention and available supports, and to normalize testing, treatment and disclosure.

*We need to normalize the fact that we need to disclose our status about whether we have contracted HIV or not, we need to normalize conversations about this between, in the community and also to address the problem of HIV (Reporter, Toronto, FG1).*

Some focus group participants spoke about the importance of critical health literacy in order to be able to advocate for themselves in the health care system and make informed decisions.

*..to change how Black people’s specific contact with health system by educating Black people in how they have to communicate with their health care providers, how to self-advocate for themselves when they go to access health care, how to have knowledge before they go to access health care (Female, Toronto, FG6).*

Several focus group participants noted that informal support systems, such as friends and family, must be involved in encouraging HIV testing and treatment.

Some participants perceived a decline in traditional ‘grassroots’ education and expressed the need to increase the availability and accessibility of HIV information in places that ACB communities frequent, such as community centers, stores, schools, and faith-based organizations.

*Instead of us waiting for people to come to the agencies to access services, reach out to them, go where they are, go back to the basics of how people used to do grassroots outreach because that was working and there was the sense that currently-- AIDS service organizations or the services out there become too corporatized and going back to the basics of grassroots and that way we will be able to do a better job at doing HIV prevention and education (Female, Toronto, FG1).*

The type of features focus group participants wanted to see included targeted, creative and culturally-specific messaging.

*Are we targeting young Black males, young Black youth. Are we targeting new immigrants? Are we targeting, you know, people who are aging, right, and it has to be a different message, you know, for the different communities (Male, Ottawa, FG5).*

*We have PhDs but we don’t like to read some pamphlets or messages like that. So but when you package it into a drama, into a song, into a poem, we actually … or into something that is a … that has pictures and colourful, we will be able to consume it better than when it is a black and white five-page document. We really get tired of reading it, you know. If you package it into a nice WhatsApp message we’ll actually be able to consume it better than when you give me a three-page document (Female, Ottawa, FG6).*

Several focus group participants spoke about the importance of developing interventions and sharing information specifically targeted for ACB communities.

*So when they came to share the information, they said my name is Jack Jones, I’m a gay person and I’m a gay White man and I’m very privileged. And then they say I am on Truvada. I take Truvada every day because I can afford it so that I can protect myself from HIV and I can have sex with anyone that I want. Well for me, this person is so privileged. Why is he coming to talk to us anti-racism anti-oppression? Because he thinks because he is a gay man he is actually oppressed or has experienced oppression but already he said I find myself very … very privileged. I live in a loft, a loft in Toronto, whatever, anyway, so all that. For me I actually, I closed down. I stopped listening (F, Ottawa).*

Other focus group participants recommended the use of positive, strength-based messaging to normalize HIV and promote HIV testing as part of a regular health exam.

*So I think kind of our way of promoting testing as a strength or as a positive thing to do is perhaps more about where we have to really go. Right? Why this is good for you as opposed to ‘oh, you know, you’ve had unsafe sex and you might be positive and you don’t … ’ like all the kind of negative messages people get. That reframing things into you know, how this helps you is-- I think that’s really important to think about (Female, Toronto, FG3).*

This could include the use of positive, vocal, role models in community education and awareness initiatives to promote HIV testing and dispel myths about people living with HIV. For example, community leaders who are open about their HIV status or peer leaders.

*The young man that you brought from the Black community, I don’t remember him, but he said he was born with HIV and he was 19 years, very vibrant, he was such an inspiration. I would hear his story and say,’ you know what, I want to go and test’. Because I see someone else who is living a wholesome life and so you know what, what does it cost me? Let me go and test, I may as well still live a wholesome life like [Name 1] is or anybody else is (Male, Ottawa, FG3)..*

In addition to increasing the availability and accessibility of HIV educational materials, several focus group participants recommended expanding activities to include media/social media messaging. For example,

*Say for example like when you are going on [public transportation] or everywhere there are all these adverts which are showing ‘oh, if you have a heart issue, or if you want to stop smoking, or if you have been beaten or you know, they are encouraging people like to go and take that step. But I have never, I think I only saw once, an advert on TTC talking about HIV or even just even programming on radio or TV. There is no like a robust programming that encourages people you know, to go out there and you know, like say ‘okay, this is an issue we have in the community, everyone let’s get involved.’ (Female, Toronto, FG3).*

### “I think the options available need to be expanded” - health policies and health provider capacity-building strategies are needed to increase access to testing and care

Focus group participants identified numerous strategies to improve health provider knowledge and competency, and reduce organizational HIV testing barriers and increase HIV testing options.

Several focus group participants made recommendations for the delivery of culturally competent HIV information and care. Foremost, health providers need information and training on HIV to reduce stigma among their patients. As one focus group participant explained,

*Reducing that stigma so that all the levels of health care professionals are comfortable talking about it so that they are hearing about this information from their doctors, from their nurses, from their community workers (Reporter, Ottawa, French, FG1).*

Another focus group participant highlighted an approach currently underway in the United States where health providers engaged patients in in-depth discussions about HIV and health.

*If you test negative, there should still be a conversation with your health care provider in terms of okay, so why did you come here. What was your exposure? You came for a reason, right. So you have that conversation about preventing HIV in the future, even if you test negative, right. So health provision should be aware of that and that is the place where you can have these conversations around PrEP, around PEP and all of these things that we’re talking about. So even if you’re testing negative, you’re assessing, you know, somebody’s you know, future risks and future options and having that conversation. Not that you test negative so go home. Everything is good. Which is what we normally do, and we focus on the person testing positive, right (Male, Ottawa, FG5).*

There was general consensus among focus group participants that access to HIV testing needs to be greatly expanded to address intersectional issues and increase availability to demographically and geographically diverse ACB communities.

*Whether you’re targeting young Black youth, or you know, older ACB folks, whether they’re first generation or have been here for a long time. So even in the ACB community, we have to have different testing strategies based on the particular demographic that you’re trying to target. And also, you have to go geographically to where people are to make it more accessible (Male, Ottawa, FG2).*

*Taking the HIV testing into some of the African, Caribbean, Black events. So we need to work proactively, taking the services closer to where our clients are. I think that helps us to remove some of the barriers and help our community members access our culturally appropriate services (Male, Toronto, FG6).*

Several focus group participants were in favour of increased access to self-testing in Canada to reduce some of the fear and stigma associated with accessing HIV testing. As one focus group participant explained,

*So, you know, the options available are rapid test, going to a clinic or going to a community health center, I think we also need to expand on the types of options available for testing, like having home kits, so that there are barriers to actually going to a physical space to get a test for some people and by offering a mail, a test that you can receive in the mail at your home that might be an option for some people and maybe a better option to maintain confidentiality and reduce stigma and things like that (Female, Ottawa, FG2).*

Many focus group participants reported that their health care providers had never discussed PEP or PrEP with them. Clearly, more training is required for health providers on these treatments, especially for those working in primary care or first point of contact services.

*So I think there’s a lot of education that needs to be done with primary care physicians and people who are offering-- or doing the tests themselves. I think that that community, the physicians, need additional opportunities for education themselves because they’re not-- they may not be exactly aware of what’s available (Female, Ottawa, FG5).*

*Unless you’re involved in work in HIV and STIs, if you go to a general doctor, GP, unless they’re a specialist, they probably will not present all the options available and the information because they may not be aware of it, because they’ll have to look it up on their computers; right? (Female, Ottawa, FG5).*

### “We are going to have our own organizations but we have to have allies” – successful strategies need to be intersectoral

There was a general recognition among focus group participants that collaborations are key to addressing educational and service related gaps, including between ACB and non-ACB, HIV and non-HIV, and health and community/school provider groups.

Focus group participants recognized that partnerships with other non-ACB providers are important to supplement the services and interventions provided by ACB communities because resources in the ACB community are often limited. As one focus group participant explained,

*We are going to have our own organizations but we have to have allies. We have to have the people with the money, the people who are making decisions as our allies so that we can work together and we are going to tell them how they are going to treat us and how they are going to work with us. So allies are going to be very important, much as we will have our own organizations but if we put … if we have organizations and we are putting ourselves in a corner and we are excluding people who actually have the resources. So how can we develop a circle of care so that our allies understand us and they work with us because we cannot all be Black people … all Black doctors, all Black nurses, all Black social workers (Female, Ottawa, FG6).*

Focus group participants described the need for collaboration between ACB community agencies (i.e., non-HIV) and HIV agencies specifically serving people at risk or living with HIV, for the provision of HIV information and support.

*I think we could build more partnerships. Maybe we need to build more partnerships with people who we don’t know. So non-HIV organization and HIV sector organization and bring them into this partnership. And do the work together (Female, Toronto, FG6).*

*So I know that, for example, sometimes there’s somebody at Malvern Health or Lawrence or wherever that are tested positive or want information about HIV or have a partner that is positive and they’re not willing to go down to ASO. So having the ASO staff goes out to those places. I think that works (Female, Toronto, FG6).*

In addressing educational needs, especially among the school-aged population, focus participants identified the school system as a preferred venue for promoting information on HIV to students and educators, but this needs to be done in collaboration with ACB community agencies. This is evident in the following quotes:

*Can you guys team up with the school system to do prevention with the little kids out in the community because not all of them go to churches...? The best way to reach them is probably to become an advocate in one of those school boards. Then you can have a team going through school system and talking to them. I think that’s the best way to reach the young kids. (Male, Toronto, FG2).*

*We should also target to have probably within the curricular, at the university level, like probably Education 101, so that we get to sensitize people. So health education or health and social education is more in schools (Male, Ottawa, FG5).*

The success of these types of initiatives, however, were largely dependent on the receptivity of individual school principals.

*I think that’s what Public Health is trying to do. There’s some schools where it’s very easy to have Black CAP come in with, you know, with us, or even on their own … and on their own depending on the principal. You know, principals are really gatekeepers of who gets to come into the school. So if they get a Public Health endorsement, they’re usually, you know, that ups the ante for them. It’s just the way the system works (Female, Toronto, FG6).*

It was widely acknowledged that intersectionality must be considered in the development and implementation of all HIV strategies. As a focus group participant explained,

*Black people organize in different ways, and I think that’s something that [Name 4] was talking about is about our own experience when we come here. If I look at Facebook groups and other places, we do organize in different ways. We organize religious … religiously, we organize, socially in like in coming from different country. So we have to really go back to how we socialize because Black people are diverse. We can’t just be one heterogeneous group and we are seen as this Black. We have to really clearly look at those social organizations within Black people, within our own diversities and ensure that this knowledge goes there (Female, Toronto, FG6).*

## Discussion

Our findings support previous research in this area finding that HIV-related stigma increases vulnerability to HIV infection by reducing access to HIV prevention, testing, and presenting barriers to treatment, care, and support for people living with HIV [30]. ACB community members continue to face stigma as a result of assumptions that HIV/AIDS is an epidemic brought to Canada by the “outsiders”/“foreigners” and is therefore an ACB people’s disease; associating the epidemic to stigmatized sexual behaviors such as sexual partner concurrency, extramarital sex, sex work, and drug use [34]. However, preventive initiative may also consider that many infections occur when individuals are unaware of their partner’s HIV status [35–37].

Findings further suggest that provider-related issues, including lack of information and training, and organizational issues, such as systemic racism continue to represent major barriers to HIV testing and treatment. HIV prevention efforts have historically focused on individual behaviours that increase HIV transmission [38]. Access to accurate HIV testing is crucial in order to direct people living with HIV to treatment programmes and those who are HIV-negative to appropriate prevention strategies. WHO [39] recommended that HIV testing and counselling processes be streamlined into primary care as these are prerequisites for critical life-saving interventions. Newman et al. [40] also identified the importance of making HIV prevention a routine part of general education and women’s health care.

Current research demonstrates that interventions, such as a daily oral PrEP, is effective in reducing the risk of sexual HIV transmission among heterosexual men and women [15–17]. PrEP cost is partially or fully covered by private and most provincial/territorial health insurance plans [17]. While PrEP use has increased with its inclusion in publicly funded drug plans (e.g. Ontario) [17, 18]. PrEP awareness and access among populations at risk of HIV varies. Among clinical service providers, the lack of education and training about PrEP is a major barrier since many do not feel prepared to discuss PrEP with patients [41, 42]. Organizational policies concerning the coverage of PrEP and PEP costs may influence eligibility criteria.

The majority of Canadian research on population awareness and willingness to use PrEP has been conducted among gay men and other MSM, with few studies looking at other at-risk populations, such as ACB communities. A review of Canadian PrEP research noted that service providers who work with ACB communities reported a low PrEP awareness in these groups [17]. This is concerning since prior awareness is needed in order for individuals at high risk of HIV exposure requires to access PrEp. Health providers’ lack of experience working with Black communities is another barrier to the provision of PrEP services to ACB patients [19]. Nelson et al. [19] found that Canadian PrEP guidelines are not sensitive to the parameters surrounding HIV transmission among ACB communities. Front-line service providers working with priority populations (ACB and MSM communities) have expressed concerns related to gender-based inequities in access to PrEP and the lack of consensus and guidance in PrEP messaging [43]. New models of care are emerging for ACB women. For example, O’Byne et al. [38], demonstrated the effectiveness of a nurse-led two-dimensional risk assessment procedure in Ottawa for persons in high prevalence groups. These authors recommended that future research focus on not only increasing access to healthcare and PrEP medications, but also on interventions for patients to appreciate the utility of PrEP for their HIV prevention [38].

Our findings support previous research on the importance of involving ACB communities as key collaborators in HIV prevention, treatment [44–46]. For example, Newman et al. [40] and other researchers, describe HIV prevention opportunities that capitalize on existing community institutions and strengths, such as working with faith leaders, facilitating access to female-controlled prevention methods (e.g., medical interventions as microbicides and antiretrovirals for post-exposure prophylaxis, self-protection methods e.g. female condom), promoting economic empowerment and independence, etc. [47, 48].

Our research further supports the adoption of peer-led approaches to meet community needs. Peer interventions select individuals who share demographic characteristics (e.g., age or gender) or risk behaviors with a target group (e.g., commercial sex work or intravenous drug use (IDU)) and train them to increase awareness, impart knowledge and encourage behavior change among members of that same group. Peer education programs are based on the rationale that peers have a strong influence on individual behaviour [49]. As members of the target group, peer educators are assumed to have a level of trust and comfort with their peers that allows for more open discussions of sensitive topics [48]. Peer education can be delivered formally in highly structured settings (such as classrooms) or informally during the course of everyday interactions. A meta-analysis on the effectiveness of peer education programs for HIV prevention in developing countries found that peer education can be an effective strategy for changing behavior among hard-to-reach, hidden populations such as commercial sex workers and IDUs [50]. Peer education programs have been shown to be empowering to both the educator [51], and to the target group by creating a sense of solidarity and collective action [52].

Critical health literacy was identified as an important component of educational and awareness strategies. Critical health literacy is increasingly being used to filter relevant messages from the current sea of information to inform personal behavioural actions and promote health especially in the current context of COVID-19 and other co-morbidities like HIV [53]. To date, the great majority of health literacy interventions have been conducted in clinical settings, and have focussed on task-directed, functional health literacy. Relatively few reported interventions incorporating the concepts of interactive and critical health literacy [54]. A review of a wide range of community-based HIV and health literacy interventions provided qualitative and quantitative evidence of positive outcomes, primarily changes in knowledge [55, 56]. Poor health literacy in the context of HIV impacts on greater difficulty in avoiding HIV infection, failing to fully understand diagnostic information, a poorer working knowledge of HIV and its treatment; not adhering to antiretroviral therapy (ART), difficulties with healthcare instructions and directions, and a greater likelihood of having a detectable viral load [57]. The highest levels of evidence were found for interventions that had high levels of community involvement at multiple stages, including active participation in evaluation processes, focus on issues such as empowerment^82^ and competence-building and including peer support [58].

This study has strengths and limitations. Among its strengths is that it was designed and led by ACB-community researchers and worked with several ACB community partners to engage ACB community members in designing the study and in generating and interpreting the research findings. Some of the limitations are common to all qualitative research. While the FGD’s were held in two Canadian centres, conducted in English and French and included a diverse sample, findings cannot be generalized to all ACB community members living in Canada. Furthermore, due to the small sample size, intersectionality considerations could not always be explored.

## Conclusions

This study was conducted to better understand barriers and facilitators to HIV-testing and treatments and to engage ACB community members in the identification of promising community and health system strategies. We learned that multi-level factors are persistent barriers to HIV testing and treatment. At the individual- and community-levels, fear, prevailing health beliefs, stigma and lack of access to culturally informed information, continue to represent major barriers to testing and care. Health system barriers included lack of provider awareness, issues related to cultural sensitivity and confidentiality and cost, and racism in the health care system. In terms of implications for health policy, study findings suggest the need to address structural and institutional factors that contribute to vulnerability including, poverty, racism, and barriers to accessing HIV information, resources, health care and supports for persons living with HIV, recognize and address policies that perpetuate stigma and discrimination including, race-based, gender, homophobia and transphobia, enable the collection of race-based data to identify and address health inequities and reduce funding barriers to evidence-based HIV prevention treatments (e.g., PEP, PrEP).

The results can be used to support ACB advocacy and activism related to health and wellbeing, help ACB communities exercise power and control over their access to health services, strengthen health programs and services, and inform programs and research in Ontario. Critical in this regard is the engagement of policy makers and health decision makers in working to “end” HIV.

## Data Availability

The data are available from the corresponding author upon reasonable request.
